# Antimicrobial Peptides from Porcine Blood Cruor Hydrolysates as a Promising Source of Antifungal Activity

**DOI:** 10.3390/foods14010008

**Published:** 2024-12-24

**Authors:** Sara García-Vela, Aurore Cournoyer, Zain Sánchez-Reinoso, Laurent Bazinet

**Affiliations:** 1 Department of Food Science, Université Laval, Québec, QC G1V 0A6, Canada; sara.garcia-vela.1@ulaval.ca (S.G.-V.); aurore.cournoyer.1@ulaval.ca (A.C.); zain.sanchez-reinoso.1@ulaval.ca (Z.S.-R.); 2 Laboratoire de Transformation Alimentaire et Procédés ÉlectroMembranaires (LTAPEM, Laboratory of Food Processing and Electromembrane Processes), Université Laval, Quebec, QC G1V 0A6, Canada; 3 Institute of Nutrition and Functional Foods (INAF), Université Laval, Quebec, QC G1V 0A6, Canada

**Keywords:** porcine cruor hydrolysates, antimicrobial peptides, antifungal peptides

## Abstract

Porcine blood, a significant byproduct of the pork industry, represents a potential source of antimicrobial peptides (AMPs). AMPs offer a promising alternative to chemical antimicrobials, which can be used as natural preservatives in the food industry. AMPs can exhibit both antibacterial and/or antifungal properties, thus improving food safety and addressing the growing concern of antibiotic and antifungal resistance. The objective of this study was to evaluate the antimicrobial activity of potential AMPs previously identified from porcine cruor hydrolysates. To this end, a total of sixteen peptides were chemically synthesized and their antimicrobial activities (antibacterial, anti-mold, and anti-yeast) were evaluated using microtitration and agar well diffusion methods against a wide range of microorganisms. Five new peptide sequences demonstrated antifungal activity, with Pep5 (FQKVVAGVANALAHKYH), an alpha-helix peptide, exhibiting the most promising results. Pep5 demonstrated efficacy against nine of the eleven fungal isolates, exhibiting low minimum inhibitory concentrations (MICs) and a fungicidal effect against key spoilage fungi (*Rhodotorula mucilaginosa*, *Debaryomyces hansenii*, *Candida guilliermondii*, *Paecilomyces* spp., *Eurotium rubrum*, *Mucor racemosus*, *Aspergillus versicolor*, *Penicillium commune*, and *P. chrysogenum*). These findings illustrate the potential of porcine blood hydrolysates as a source of AMPs, particularly antifungal peptides, which are less known and less studied than the antibacterial ones. Among the tested sequences, Pep5 exhibited the most promising characteristics, including broad-spectrum activity, low MICs, and a fungicidal effect. It is, therefore, a promising candidate for further research and for potential applications in the porcine industry and beyond.

## 1. Introduction

Antimicrobial resistance (AMR) is a serious public health problem that affects the treatment of human and animal infections and is associated with the unnecessary prescription and/or misuse of antibiotics. Antimicrobials are widely used in human and veterinary medicine and animal husbandry, and in some areas as growth promoters, although they have been banned in Europe since 2006 [1]. Antimicrobial resistance is developing rapidly and includes both antibacterial and antifungal resistance. The misuse and overuse of antibiotics creates a selective evolutionary pressure that leads to increased resistance [2,3], which can be spread rapidly by bacteria through genetic recombination within and between species. Antibiotic resistance primarily involves bacteria developing mechanisms to evade the effects of antibiotics, which can include enzymatic degradation of the drug, alteration of drug targets, and increased drug efflux from the bacterial cell. This phenomenon is well documented and the World Health Organization (WHO) has identified antimicrobial resistance as one of the top ten global health threats [4]. While to a lesser extent, due to their genetic plasticity and versatility in homeostatic responses to stressful environmental cues, fungi can also develop multiple mechanisms of resistance to antifungal drugs, which is an equally pressing issue [5]. Its importance is reflected in the increase in the prevalence of invasive fungal infections and their associated mortality rates [6,7]. Moreover, it also threatens food security and ecosystem health, as fungal pathogens reduce agricultural productivity [8]. Overall, inappropriate use of antimicrobials contributes to the emergence and spread of resistance. Resistant microorganisms can be transmitted to humans through the food chain and water, or through contact with animals. For this reason, the WHO has proposed to address this issue from a One Health perspective through the development of new alternatives to antimicrobial use in livestock and agriculture [5,9].

While the establishment of appropriate antibiotic usage patterns represents a pivotal strategy for mitigating the spread of antibiotic resistance, the identification of novel alternatives is of crucial importance. Promising strategies include the utilization of bacteriophages, vaccines, antibodies, and phytochemicals, enzyme preparation, probiotics, plant extracts, among others [10,11,12]. The development of novel antifungal compounds is essential not only to improve food safety but also to address the growing concern of antifungal resistance. Diversifying antifungal strategies by incorporating new antifungal compounds is crucial [13,14]. Examples of novel approaches include the study of fatty acids and oxylipins to inhibit fungal growth and mycotoxin production [15], or the incorporation of bioprotective cultures, such as *Lactobacillus* strains, into food products, which can extend shelf life while providing a safer alternative to synthetic preservatives [16]. Consequently, AMPs represent a promising new source of antimicrobial properties [17]. These small, cationic peptides are ubiquitous in nature and exhibit enormous structural diversity across all kingdoms of life. Their increasing study is a prominent area of research [18], with over 3100 natural AMPs identified from bacteria, fungi, plants, insects, fish, birds and other animals. They are considered to be effective replacements for traditional antimicrobials, particularly due to their potent, broad-spectrum antibacterial and antifungal activity and the difficulty for pathogens to develop resistance to them [19,20,21].

Conversely, porcine blood, a significant byproduct of the slaughter process, serves as a protein source from which AMPs can be extracted. This provides an original source of AMPs while promoting the use of a circular economy [22]. After centrifugation of porcine blood, two different fractions are obtained. The first is plasma, which contains 6% to 8% proteins, including albumin, globulins and fibrinogen. The second fraction, commonly known as the cruor or cellular fraction, contains the formed elements of the blood. These include mainly red blood cells (erythrocytes), which contain hemoglobin [23,24,25]. Due to its high protein content, mainly hemoglobin (90% of the protein), cruor is an optimal substrate for proteolysis [17,18,19,20,21,22,23,24,25,26,27]. Antimicrobial peptides have been successfully extracted from the peptide hydrolysate of bovine cruor as it has been the subject of extensive investigation. In parallel, although not extensively studied, porcine cruor hydrolysates have recently been shown to be a source of bioactive peptides [22,28]. Overall, porcine cruor hydrolysates could represent a source of natural antibacterial and/or antifungal activity, where novel peptides could be identified with potential applications in the food industry, in an eco-circular context of reusing this by-product to increase the shelf-life of food products.

In this context, this general goal of the present study is to contribute to the field of AMPs research by identifying new AMPs sequences with antimicrobial activities (antibacterial, anti-yeast, and/or anti-mold) against relevant microorganisms affecting the food industry, thus highlighting their potential use as food preservatives. Hence, the research objective of this study was to assess the antimicrobial activity of peptide sequences previously identified, but not demonstrated as antimicrobial sequences, in porcine cruor hydrolysates. Specifically, this study aimed to: (1) determine the antibacterial and antifungal (anti-yeast and anti-mold) potential of these sequences, (2) investigate the possible secondary structure of promising active sequences, (3) identify common motifs among them, and (4) establish whether there is synergy between active sequences.

## 2. Materials and Methods

### 2.1. Peptide Sequences

Sixteen new peptide sequences, not reported in the literature for antimicrobial activity, were selected for this study, previously identified from porcine cruor hydrolysed in different conditions [22,29,30]. The physicochemical properties of the selected peptides calculated according to [31] amino acid chain length, molecular weight, isoelectric point and GRAVY index, which are summarized in Table 1. Pep1 to Pep9 were identified in porcine cruor hydrolysed using pepsin at pH 3, 23 °C and 30 min [22], which were further separated by electrodialysis with ultrafiltration membrane [29,30]. In addition, Pep10 to Pep15 were identified from porcine cruor hydrolysed with pepsin at two different temperatures (23 °C and 37 °C) for 3 h [32]. These peptides were selected based on peptide abundance observed in the porcine cruor hydrolysates and on their antimicrobial activity predicted by bioinformatic tools [33,34].

The sixteen peptide sequences were synthesized by Synpeptide Co., Ltd. (Shanghai, China) using the standard solid-phase peptide synthesis (SPPS) on an automated peptide synthesizer [35], with a purity > 90%. Briefly, for the SPPS, a 2-chlorotrityl chloride resin (MilliporeSigma Canada Ltd., Oakville, ON, Canada) was used and Fmoc strategy was employed for deprotection. Final purification was performed using liquid chromatography.

All synthesized sequences were diluted in 10% DMSO H_2_O (Bio Basic, Markham, ON, Canada) (*v/v*), reaching a stock concentration of 2.5 mM and stored at −20 °C [22].

Additionally, multiple sequence alignments were generated using MUSCLE [36] via the Job Dispatcher platform from the European Bioinformatics Institute (EMBL-EBI). The 3D structure of a relevant sequence was modeled using the I-TASSER service with default parameters [37,38,39].

### 2.2. Strain Collection and Mantainance

A first screening for antimicrobial activity of the peptides was carried out using the following indicator microorganisms: the Gram-negative *Escherichia coli* MP 4100, the Gram-positive *Listeria ivanovii* HP B28, the yeast *Rhodotorula mucilaginosa* 27173, and the mold *Paecilomyces* spp. 5332-9a. *E. coli* was selected since it is a Gram-negative common foodborne pathogen, whereas *L. ivanovii* was chosen as Gram-positive strain to provide insights into the prevention of listeriosis, a significant concern in certain food products. The mold (*Paecilomyces* spp.) and the yeast (*R. mucilaginosa*) were chosen since they are commonly associated with food spoilage.

Peptides exhibiting antifungal activity were then tested against a wider fungal strain collection, including the yeasts *Candida parasilopsis* 27167, *C. guilliermondii* 27168, *Debaryomyces hansenii* LL11042 and the molds *Eurotium rubrum* 3071.14a, *Mucor racemosus* LMA-722, *Penicillium chrysogenum* LMA-212, *P. commune* 27163, *Aspergillus niger* ATCC1015, and *A. versicolor* LMA-370. All strains belong to the University Laval collection, department of Food Science (Québec, QC, Canada). These strains were selected due to their relevance in spoilage and food safety [40].

Tryptic Soy Agar (TSA) (MilliporeSigma Canada Ltd., Oakville, ON, Canada) was used for the growth and maintenance of bacterial isolates, while Potato Dextrose Agar (PDA) (BD Difco Laboratories, Sparks, MD, USA) was used for fungal isolates. For media preparation, manufacturer instructions were followed, and 1.0% agar was added to Tryptic Soy and Potato Dextrose culture media. Incubation conditions were as follows: 37 °C for 24 h for bacterial strains, 25 °C for 48 h for yeast strains, and 25 °C for 4–5 days for molds, all under aerobic conditions.

### 2.3. Antimicrobial Activities

Agar well diffusion method and microtitration methods were used to evaluate the antimicrobial activities of the sixteen peptides against the indicator strains collection. In order to evaluate the potential usage of two promising antifungal peptides, the checkerboard assay was used to elucidate their synergistic/antagonistic relationships. All assays were performed in triplicates.

#### 2.3.1. Agar Well Diffusion

The sixteen peptides were initially tested using the agar well diffusion method against the indicator strains *E. coli*, *L. ivanovii*, *R. mucilaginosa*, and *Paecilomyces* spp., as previously described in [22]. To perform this procedure, 25 µL of the prepared culture suspension (adjusted to 10^6^ CFU/mL for bacteria and yeasts and 10^5^ CFU/mL for molds) was diluted in 25 mL of Tryptic Soy Broth (TSB) (for bacterial strains) or Potato Dextrose Broth (PDB) (for fungal strains) supplemented with 0.75% agar and poured into a Petri dish (90 mm diameter) until dried. The culture media of the suspension was prepared using TSB for bacterial cultures and peptone water (10% *w*/*v*) supplemented with 0.1% Tween 80 (*w*/*w*) for fungal cultures as it enhances spore dispersion. Subsequent to the drying process, wells were formed using a sterile 5 mL pipette. A volume of 80 µL of each peptide at a concentration of 2.5 mM was added to each well. Ampicillin (MilliporeSigma Canada Ltd., Oakville, ON, Canada), at a concentration of 256 µg/mL and natamycin (MilliporeSigma Canada Ltd., Oakville, ON, Canada) at 50 µg/mL, were used as positive controls for bacterial and fungal tests, respectively, as they are wide-spectrum antimicrobials. The solvent of the peptides was included as negative control in all experiments. Finally, the agar plates were incubated at 37 °C for 24 h for bacterial tests and at 25 °C for 48 h for fungal tests. Thereafter, inhibition halos were measured, and images were captured using a Biorad Chemidoc camera.

#### 2.3.2. Minimum Inhibitory Concentration (MIC) and Minimum Bactericidal/Fungicidal Concentration (MBC/MFC)

A microtitration assay was conducted to determine the minimum inhibitory concentration (MIC) of the peptides against the collection of indicator microorganisms [22]. The growth medium used for the isolated bacterial and fungal strains was TSB and PDB, respectively. In brief, 175 µL of the culture medium was added to the wells of column 1 (negative growth control) and 125 µL to the wells of columns 2–12. Subsequently, 125 µL of each peptide (stock concentration of 2.5 mM), positive control (stock concentration of 256 µg/mL for ampicillin in bacterial tests and 50 µg/mL for natamycin in fungal tests) and negative control (10% DMSO in H_2_O (*v*/*v*)) were added to the wells in column 3 and mixed by pipetting up and down 10 times. A total of 125 µL from column 3 was removed and transferred to column 4, where serial dilutions were performed until the final dilution reached column 12. The final volume was then discarded. Thus, the peptide concentration in each well starts from 1.25 mM and decreases in 1:2. Subsequently, 50 µL of the indicator strain suspension (at a concentration of 10^5^ CFU/mL for yeasts and bacteria and 10^4^ CFU/mL for molds) was introduced to all the wells, except for column 1. The culture medium used for culture suspension was identical to that employed in the agar well diffusion method. The microplate was incubated for 24 h at 37 °C for bacterial isolates and for 48 h at 25 °C for fungal isolates. Following the incubation period, the number of wells exhibiting inhibition was recorded by measuring the optical density at 595 nm using a PowerWave XS2 microplate reader (BioTek Instruments, Winooski, VT, USA) and the program Gen5 2.09.

The minimal bactericidal/fungicidal concentration (MBC/MFC) was calculated by transferring 10 µL of the wells where no growth could be detected to agar plates (TSA for bacterial isolates and Dichloran Rose Bengal Chloramphenicol Agar (DBRC)) (BD Difco Laboratories, Sparks, MD, USA). After incubation (24 h, 37 °C for bacteria and 48 h, 25 °C, for fungi), the peptide concentration with which no growth was observed, was considered to be the MBC/MFC. The MBC/MIC and MFC/MIC ratios were calculated in order to ascertain the effect of the peptides. A ratio of ≤4 is indicative of bactericidal/fungicidal efficacy, whereas a ratio of >4 indicates bacteriostatic/fungistatic activity.

#### 2.3.3. Checkerboard Assay

In order to evaluate synergistic/antagonistic relationships between active peptides, the fractional inhibitory concentration (FIC) index (a quantitative measure used to evaluate the interaction between two antimicrobial agents) was calculated using the microdilution checkerboard method, as previously indicated [41]. In summary, based on the MIC values of each selected peptide (Pep5 and Pep4) for a given strain, a twofold serial dilution of compound A (Pep5) was prepared horizontally in a 96-well microtitre plate, starting at a concentration 32 times its MIC. Similarly, compound B (Pep4) was diluted vertically in a separate plate, starting at eight times its MIC. Then, 50 μL of the compound B dilutions were added to the wells of the microplate containing compound A. Finally, 50 μL of a cell culture of *R. mucilaginosa* (10^5^ CFU/mL) was added to the microplate and the plates were incubated under ideal growth conditions: 24 h, 25 °C in aerobiosis [42].

The FIC index was calculated as follows [43]:

FIC index=FIC of drug A + FIC of drug B

where

$$FIC\;of\;drug\;A\;=\;\frac{MIC\;of\;A\;alone}{MIC\;of\;A\;in\;combination}$$

$$FIC\;of\;drug\;B\;=\;\frac{MIC\;of\;B\;alone}{MIC\;of\;B\;in\;combination}$$

The effect of the different combinations was interpreted as follows: FIC ≤ 0.5 for a synergetic effect, 0.5 < FIC ≤ 0.75 for partial synergy, 0.75 < FIC < 1 for additivity, 1 ≤ FIC ≤ 4 for neutrality, and FIC > 4 for antagonism.

## 3. Results

### 3.1. Antimicrobial Activity of the Synthetized Peptides

**Figure 1:**
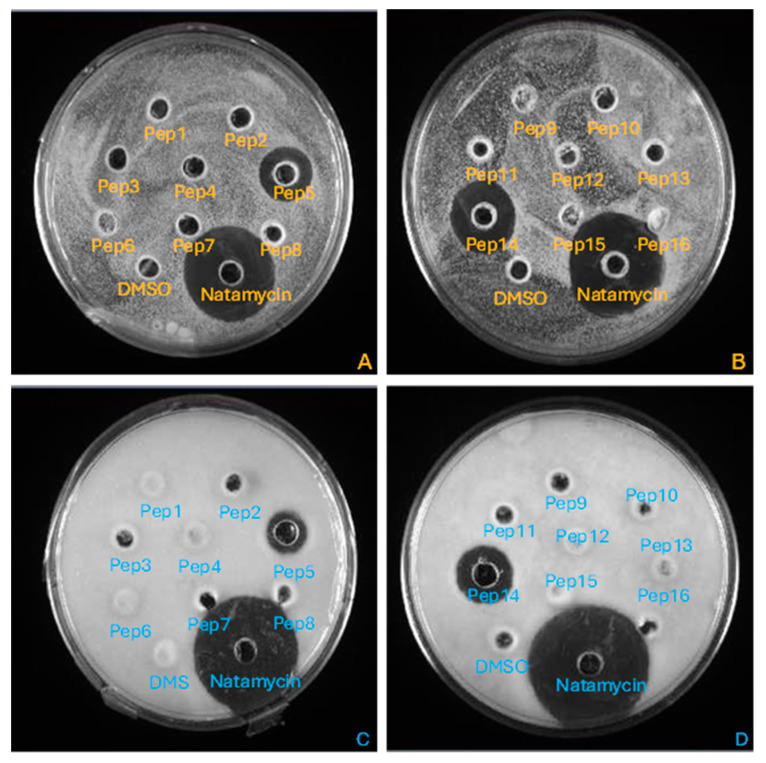
Inhibition halos of the peptides against *R. mucilaginosa* (pictures (**A**,**B**)) and *Paecilomyces* spp. (pictures (**C**,**D**)). Clear inhibition halos, as observed for Natamycin, Pep5, and Pep14, indicate antimicrobial activity.

The agar diffusion method demonstrated the inhibitory effects of only Pep5 and Pep14 against *R. mucilaginosa* and *Paecilomyces* spp., as indicated by the formation of distinct inhibition halos (Figure 1). The diameter of the inhibition halos was as follows: for Pep5; 21 mm and 19 mm against *R. mucilaginosa* and *Paecilomyces* spp., respectively, and, for Pep14; 23 mm and 20 mm against *R. mucilaginosa* and *Paecilomyces* spp., respectively.

The MICs and MFCs of the sixteen peptides against *R. mucilaginosa* and *Paecilomyces* spp. are presented in Table 2. The MIC and MFC/MIC ratio indicated that five peptides exhibited antifungal activity against the fungal isolates, with fungicidal effects. None of the sixteen peptides was active against bacterial isolates.

**Figure 2:**
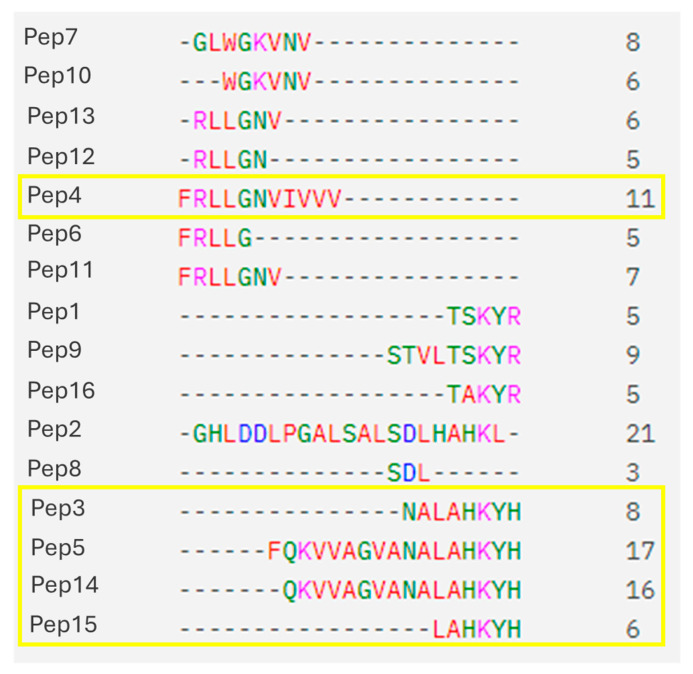
Mapping of the pairwise peptide alignment from the sixteen peptides tested. Numbers on the right indicate the number of amino acids in each peptide. Sequences highlighted by yellow squares indicate active sequences.

Pep4 (MIC = 0.375 mM against *R. mucilaginosa* and MIC = 0.625 mM *against Paecilomyces* spp.) and Pep5 (MIC = 0.039 mM against *R. mucilaginosa* and MIC = 0.009 mM against *Paecilomyces* spp.) were selected for further analysis due to their high inhibitory activity and the notable disparity in their amino acid sequences (Figure 2). The MICs and MFCs were calculated for these two peptides against a broader collection of fungal strains. The results are presented in Table 3. As indicated in Table 3, Pep5 exhibited the lowest minimum inhibitory concentrations (MICs) and the widest spectrum of activity.

Overall, the methodology employed did not yield any evidence of antibacterial activity in the sixteen peptides that were tested. Conversely, antifungal activity was observed for five of the sixteen peptides.

### 3.2. Synergistic Effect Between Pep4 and Pep5

The checkerboard assay did not reveal any evidence of synergy between Pep4 and Pep5 against *R. mucilaginosa* or *Paecilomyces* spp. Indeed, growth was observed in all wells (with the exception of the controls). Thus, the FIC index could not be calculated. But, since growth was observed in all wells, the FIC index should be >4, indicating that their relationship is antagonistic.

### 3.3. Pep5 3D Structure

**Figure 3:**
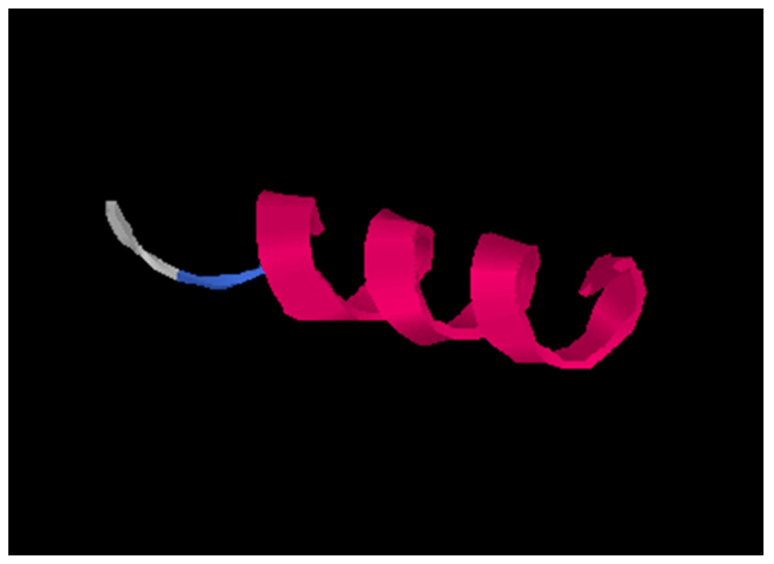
Alpha-helix secondary structure of Pep5. Amino acid sequence: FQKVVAGVANALAHKYH; molecular weight 185.16 g/mol and GRAVY index 0.20. Image generated by I-TASSER server.

Due to its strong antifungal activity, Pep5 secondary structure was predicted, which resulted in an alpha-helix (Figure 3). The remaining active peptides are not represented due to the fact that they are composed of shorter chains which often are not suitable for predictions.

## 4. Discussion and Future Perspectives

Sixteen new sequences previously identified from porcine hydrolysates were analyzed for antimicrobial activity in this study. Antifungal activity was found for five of them, whereas no antibacterial activity was detected (Table 2). In previous studies, the porcine hydrolysates from which these sequences were identified showed both antibacterial and antifungal activity [22,29,30]. A possible explanation for this could be that the peptides present in the hydrolysate may have interactions between them that result in antibacterial activity, whereas each of them individually does not. Another possibility is that, among all peptides contained in the hydrolysates, the selection of sequences for chemical synthesis, yielded only antifungal activity, while other sequences, also present in the hydrolysates, which have not been chosen for the current research, could yield antibacterial activity. Nevertheless, antifungal activity is not commonly studied in AMPs and the development of new antifungal compounds is crucial in the food industry. Traditionally, chemical antifungal preservatives such as sorbates and propionates have been widely used. However, regulatory changes have limited their concentrations and alternative methods need to be explored [44]. In this line, the use of AMPs coming from cruor hydrolysates could represent an organic and clean-label alternative. In addition, the prevalence of fungal contamination of food products is increasing. This poses significant health risks and economic losses. Fungal spoilage not only affects food quality but also leads to the production of mycotoxins, which are harmful for human health [13]. The limited number of antifungals currently available highlights the urgent need for innovative solutions in food preservation [13,14]. Alternatives to traditional antifungal preservatives include the use of bioprotective cultures as well as the use of fatty acids and oxylipids [15,16]. However, the use of bioprotective cultures requires extensive research regarding safety concerns, given that they are living microorganisms. In contrast, AMPs could represent a safer option. The results of this research shed light on this pressing issue, demonstrating the potential of porcine cruor hydrolysates as a source of AMPs and revealing five novel antifungal peptide sequences that could be used as food preservatives for porcine products and beyond [45].

Of all the sequences tested, five showed antifungal activity by microtitration assays (Table 2), while only two showed a clear inhibition halo (Figure 1). This could be explained by the fact that the agar well diffusion method relies on the ability of the antimicrobial compound to diffuse through the agar medium. Compounds with low diffusivity due to molecular size, hydrophobicity, or binding affinity to agar components may not diffuse sufficiently to produce visible inhibition zones [46]. Furthermore, the microtitration method is generally more sensitive than the agar diffusion method. It can detect lower concentrations of antimicrobials which inhibit microbial growth without necessarily producing a visible halo [47]. Thus, five of the sixteen sequences are considered antifungal, even though only two peptides showed clear inhibition halos.

Among the sequences tested, the antibacterial compound Pep1 (TSKYR), previously described as having a broad spectrum of activity [28,48,49,50], was found to lack antimicrobial activity when tested using the methodology employed. This could be explained by the way in which this sequence was obtained or chemically synthesized; in previous studies, it was obtained in some cases by enzymatic hydrolysis or chemical synthesis.

Among the antifungal compounds, Pep5 exhibited a MIC values as low as 0.009 mM and 0.039 mM for *Paecilomyces* spp. and *R. mucilaginosa*, respectively. MFC/MIC ratios revealed fungicidal effect on the active peptides, making them very efficient for fungal control. Pep5 and Pep4 were chosen for further analysis. Pep5 was chosen for being the most active peptide with a strong fungicidal effect. Conversely, Pep4 was chosen even though its MICs were not as low as other active peptides because it was active while exhibiting a completely different sequence to that of Pep5 and the other active peptides (Figure 2). These peptides were tested against a wider collection of fungal strains including the yeasts responsible for the spoilage of meat products (including pork products), such as *R. mucilaginosa* (responsible for the spoilage of salt-cured meat), *Debaryomyces* spp., *Rhodotorula* spp., and *Candida* spp. (responsible for the spoilage of fresh meat), [51], and against relevant molds, including some species of the genera *Aspergillus*, *Penicillium*, and *Eurotium* (commonly responsible for dry and smoked meat spoilage,) as well as *Mucor racemosus* (responsible for whisker-like spoilage) [51]. In all scenarios Pep5 was more active than Pep4, exhibiting lower MICs and a wider spectrum of activity (Table 3). It can be concluded that AMPs derived from porcine blood cruor can be employed to prevent mold-related spoilage of meat in the food industry. Furthermore, these peptides can be utilized, not only in the porcine industry, but in other industries due to their elevated activity against molds, where their presence represents a significant issue, such as in the bakery industry [52].

A checkerboard assay was conducted to ascertain whether a synergistic interaction exists between Pep5 and Pep4. However, the results of the assay indicated that there was no evidence of synergy between them. The FIC index could not be calculated since growth was observed in all wells of the microplate, suggesting that it should be >4, thereby indicating an antagonistic relationship.

With regard to the amino acid sequence of the AMPs, the consensus LAHKYH was identified in four of the five active sequences. A homologous sequence, LAHRYH, was previously described as an antibacterial peptide from bovine hydrolysates [48]. In porcine hemoglobin, arginine (R) is replaced by lysine (K, position 145) in the amino acid sequence. Both amino acids are positively charged, and thus the total hydrophobicity of the peptide is not affected by the modification of the arginine amino acid, as previously described in [22]. Hydrophobic amino acids such as valine, leucine, and aromatic residues contribute to AMP amphipathicity. This amphipathicity allows the peptides to separate into hydrophobic and positively charged regions, which facilitates their interactions with negatively charged fungal membranes [53]. The hydrophobic regions of AMPs tend to insert into the lipid bilayer of the fungal cell membrane, leading to membrane disruption and ultimately cell death [54]. The five active sequences detected in this study exhibited disparate hydrophobicity profiles, with Pep4 displaying the most hydrophobic characteristics (GRAVY index = 2.12). This suggests that it may interact more effectively with lipidic membranes, leading to a higher affinity and, consequently, enhanced antifungal activity. Nevertheless, despite its activity, Pep4 exhibited higher MICs than the other peptides. The antifungal activity of the peptide may be attributed to its hydrophobic nature, as it is the only peptide lacking the consensus LAHKYH. The most active peptide sequence, Pep5, along with Pep4 and Pep14, also exhibited a positive GRAVY index, indicating their potential interaction with lipidic membranes. Conversely, Pep3 and Pep15 exhibited negative values (−0.93 and −1.00, respectively). Despite their hydrophilic nature, these peptides can possess hydrophobic regions that interact with lipidic membranes, thereby generating antimicrobial activity. With respect to the sequence, Pep5 differs from Pep14 by the presence of one additional amino acid, F, which results in a twofold reduction in the MIC against *R. mucilaginosa*. This indicates that the antimicrobial activity of the peptide is dependent on the presence of phenylalanine. Moreover, this amino acid residue increased the GRAVY score (from 0.04 to 0.20), rendering the peptide more hydrophobic and, consequently, more likely to interact with the membranes [55]. This phenomenon has been previously observed [56,57], whereby an increase in hydrophobicity correlates with an increase in antimicrobial potency. However, this can also lead to an increase in hemolysis, which may limit the peptide’s safety profile. Further research on hemolysis analysis and other parameters of Pep5 will provide valuable insights into its safety profile and potential applications. On the other hand, the Pep4 sequence differs from the Pep11 sequence by having a valine-rich tail (IVVV). Valine-rich AMPs have been shown to have greater antimicrobial activity. Valine is a hydrophobic amino acid that contributes to the overall hydrophobicity of AMPs, which is essential for their interaction with lipid membranes, leading to their disruption [58,59]. It can therefore be hypothesized that the IVV in the C-terminus of Pep4 is relevant to its antifungal activity.

Among all the peptides studied, Pep5 (sequence FQKVVAGVANALAHKYH) exhibited the highest antimicrobial activity, demonstrating efficacy against nine of the eleven fungal strains tested. In order to elucidate the basis for this elevated level of activity, the prediction of its secondary structure was conducted. Secondary structure affects peptide binding, membrane rupture, and thus, antimicrobial activity [55]. It was found that Pep5 adopts an alpha helix, which is known to be an active structure of antimicrobial peptides (AMPs), among other conformations [60]. Alpha helices favor interactions with membranes, pore formation, and stability. The amphipathic nature of alpha helices, which is higher than that of disordered structures, allows AMPs to effectively target and disrupt microbial membranes, making them potent agents against a wide range of pathogens.

Further analysis of the peptide’s mechanism of action and potential structural modifications to enhance its antimicrobial activity could yield valuable insights for future research. Moreover, a significant limitation of this study is that the assays have been conducted in vitro. Further research on this peptide could be conducted to evaluate its in vivo activity and ascertain its viability as a food preservative. This could be applied to porcine meat products, as the sequence has been identified from a byproduct of this industry in the context of a circular economy. Additionally, it could be used in other types of industries, such as baked goods, where contamination by fungi represents a significant concern.

In conclusion, the present research identified five novel antifungal sequences, of which the AMP Pep5 (FQKVVAGVANALAHKYH) exhibited the strongest activity and broader spectrum. These findings suggest that Pep5 may have potential future applications in the food industry as a preservative in porcine meat, among other uses.

## Figures and Tables

**Table 1:** Physicochemical properties of the sixteen peptides synthetized from porcine blood cruor hydrolysates [22,29,30].

Name	Sequence	Length	Molecular Weight (g/mol)	Isoelectric Point	GRAVY
Pep1	TSKYR	5	653.74	9.99	−2.24
Pep2	GHLDDLPGALSALSDLHAHKL	21	2180.45	5.71	0.01
Pep3	NALAHKYH	8	953.07	8.61	−0.96
Pep4	FRLLGNVIVVV	11	1228.54	9.75	2.12
Pep5	FQKVVAGVANALAHKYH	17	1853.16	9.70	0.20
Pep6	FRLLG	5	604.75	9.75	1.10
Pep7	GLWGKVNV	8	872.03	8.75	0.39
Pep8	SDL	3	333.341	4.05	−0.17
Pep9	STVLTSKYR	9	1054.21	9.99	−0.52
Pep10	WGKVNV	6	701.82	8.75	−0.05
Pep11	FRLLGNV	7	817.99	9.75	0.88
Pep12	RLLGN	5	571.68	9.75	−0.16
Pep13	RLLGNV	6	670.81	9.75	0.56
Pep14	QKVVAGVANALAHKYH	16	1705.98	9.70	0.04
Pep15	LAHKYH	6	767.89	8.61	−1.00
Pep16	TAKYR	5	637.74	9.99	−1.72

**Table 2:** Minimum inhibitory concentration (MIC) and minimum fungicidal concentration (MFC), in milliMolar (mM), of the sixteen synthesized peptides. NI indicates no growth inhibition. ND indicates not determined. The MFC/MIC ratio is also indicated; ratios from 1 to 4 indicate fungicidal effect, while rations > 4 indicate fungistatic effects.

Name	Activity Against *R. mucilaginosa*			Activity Against *Paecilomyces* spp.		
	MIC	MFC	MFC/MIC	MIC	MFC	MFC/MIC
Pep1	NI	NI	ND	NI	NI	ND
Pep2	NI	NI	ND	NI	NI	ND
Pep3	0.625 ± 0.0	0.625 ± 0.0	1	0.625 ± 0.0	1.25 ± 0.0	2
Pep4	0.375 ± 0.0	NI	ND	0.625 ± 0.0	0.625 ± 0.0	1
Pep5	0.039 ± 0.0	0.078 ± 0.0	2	0.009 ± 0.0	0.009 ± 0.0	1
Pep6	NI	NI	ND	NI	NI	ND
Pep7	NI	NI	ND	NI	NI	ND
Pep8	NI	NI	ND	NI	NI	ND
Pep9	NI	NI	ND	NI	NI	ND
Pep10	NI	NI	ND	NI	NI	ND
Pep11	NI	NI	ND	NI	NI	ND
Pep12	NI	NI	ND	NI	NI	ND
Pep13	NI	NI	ND	NI	NI	ND
Pep14	0.078 ± 0.0	0.078 ± 0.0	1	0.078 ± 0.0	0.078 ± 0.0	1
Pep15	1.25 ± 0.0	1.25 ± 0.0	1	1.25 ± 0.0	NI	ND
Pep16	NI	NI	ND	NI	NI	ND

**Table 3:** MIC, MFC (in mM), and the MFC/MIC ratio of the two selected peptides against the collection of fungal strains. NI indicates no growth inhibition. ND indicates not determined. The MFC/MIC ratio is also indicated; ratios from 1 to 4 indicate fungicidal effect, while rations > 4 indicate fungistatic effects.

Indicator Fungal Strains	Values	Pep4	Pep5
*Candida guilliermondii*	MIC	NI	0.312 ± 0.0
	MFC	NI	0.625 ± 0.0
	MFC/MIC	ND	2
*Candida parasilopsis*	MIC	NI	NI
	MFC	NI	NI
	MFC/MIC	ND	ND
*Debaryomyces hansenii*	MIC	0.312 ± 0.0	0.156 ± 0.0
	MFC	NI	0.156 ± 0.0
	MFC/MIC	ND	1
*Aspergillus versicolor*	MIC	0.312 ± 0.0	0.039 ± 0.0
	MFC	NI	0.078 ± 0.0
	MFC/MIC	ND	2
*Aspergillus niger*	MIC	NI	NI
	MFC	NI	NI
	MFC/MIC	ND	ND
*Penicillium commune*	MIC	NI	0.312 ± 0.0
	MFC	NI	0.312 ± 0.0
	MFC/MIC	ND	1
*Penicillium chrysogenum*	MIC	0.156 ± 0.0	0.312 ± 0.0
	MFC	NI	0.156 ± 0.0
	MFC/MIC	ND	2
*Eurotium rubrum*	MIC	0.312 ± 0.0	0.039 ± 0.0
	MFC	NI	0.078 ± 0.0
	MFC/MIC	ND	2
*Mucor racemosus*	MIC	NI	0.312 ± 0.0
	MFC	NI	0.312 ± 0.0
	MFC/MIC	ND	1

## Data Availability

The original contributions presented in the study are included in the article, further inquiries can be directed to the corresponding author.
